# Effects of Rifaximin on Circulating Albumin Structures and Serum Ammonia Levels in Patients with Liver Cirrhosis: A Preliminary Study

**DOI:** 10.3390/jcm11247318

**Published:** 2022-12-09

**Authors:** Takao Miwa, Tatsunori Hanai, Kenji Imai, Koji Takai, Makoto Shiraki, Hideki Hayashi, Shogo Shimizu, Yoichi Nishigaki, Eiichi Tomita, Masahito Shimizu

**Affiliations:** 1 Department of Gastroenterology/Internal Medicine, Graduate School of Medicine, Gifu University, Gifu 501-1194, Japan; 2 Health Administration Center, Gifu University, Gifu 501-1193, Japan; 3 Center for Nutrition Support & Infection Control, Gifu University Hospital, Gifu 501-1194, Japan; 4 Division for Regional Cancer Control, Graduate School of Medicine, Gifu University, Gifu 501-1193, Japan; 5 Department of Gastroenterology, Chuno Kosei Hospital, Gifu 501-3802, Japan; 6 Department of Gastroenterology and Hepatology, Gifu Municipal Hospital, Gifu 500-8513, Japan; 7 Department of Gastroenterology, Gifu Prefectural General Medical Center, Gifu 500-8717, Japan

**Keywords:** albumin structure, ammonia, hepatic encephalopathy, oxidative stress, rifaximin

## Abstract

Circulating albumin structures, including their oxidized and reduced forms, are involved in hepatic encephalopathy (HE) development. However, the effects of rifaximin, a key drug in HE treatment, on the circulating albumin structure in patients with liver cirrhosis remain unclear. In this multicenter prospective study, eight patients with hyperammonemia (≥80 μg/dL) were enrolled. The circulating albumin structure was evaluated using the ratio of oxidized albumin (human nonmercaptalbumin, HNA). Patients were administered 400 mg rifaximin 3 times/day for 3 months, and laboratory data were assessed at baseline and during observation. Among the eight patients, three were men; the median age and body mass index were 70 years and 26.4 kg/m^2^, respectively. The median HNA and serum ammonia levels at baseline were 41% and 143 μg/dL, respectively. After rifaximin therapy, HNA showed a decreasing tendency (median; from 41% to 36%, *p* = 0.321), but serum albumin levels showed no significant change (from 3.5 g/dL to 3.5 g/dL, *p* = 1.00); serum ammonia levels significantly reduced (median: 143 μg/dL to 76 μg/dL, *p* = 0.015). Thus, rifaximin reduces serum ammonia levels and may improve circulating albumin structure in patients with cirrhosis. Further large-scale studies are required to confirm these preliminary results.

## 1. Introduction

Liver cirrhosis, the final pathological condition of chronic liver disease, accounts for 1.2 million deaths per year worldwide [1]. Approximately 400,000–500,000 patients with cirrhosis reside in Japan [2]. Annually, 10% of patients with cirrhosis develop complications such as ascites, varices hemorrhage, and hepatic encephalopathy (HE), which are critically associated with decreased health-related quality of life, frequent health care utilization, and worse survival [3].

HE is a common neurological disorder caused by liver dysfunction ranging from subclinical alterations to coma [4]. HE is the most devastating complication of cirrhosis and leads to hospitalization, readmission, and high mortality [5,6]. Liver cirrhosis causes liver dysfunction, portosystemic shunting, malnutrition, sarcopenia, frailty, electrolyte imbalance, and microbiota dysbiosis, all of which can induce HE [4,7]. These conditions may also induce hyperammonemia and systemic inflammation, resulting in astrocyte swelling in patients with HE [8,9,10]. Oxidative stress, in particular, plays a critical role in astrocyte swelling [9,10]. Multiple mechanisms exist for generating oxidative stress in patients with HE, including overexpression of N-methyl-D-aspartate receptors, activation of nicotinamide adenine dinucleotide phosphate oxidase, and shuttling of glutamine to the mitochondria [10]. Ammonia induces astrocyte senescence in an oxidative stress-dependent manner [10]. Basic studies have reported that while ribonucleic acid oxidation in astrocytes is increased in hyperammonemia rats [11], antioxidant therapy ameliorates HE in rats [12]. These findings indicate that oxidative stress is a potential treatment target for HE.

In patients with cirrhosis, alterations in the structures of circulating albumin caused by inflammation and redox changes are considered valuable biomarkers for evaluating oxidative stress [13]. Albumin contains 35 cystine residues, which contribute to the stability and long half-life of the protein, and the only free residue is located at position 34 (Cys34) [14]. In healthy adults, about 70–80% of circulating albumin have the Cys34 residue with free sulfhydryl group, whereas about 25–30% have Cys34 forming a mixed disulfide with either cysteine or homocysteine or glutathione (human nonmercaptalbumin, HNA) [15], whereas the ratio of HNA is around 40% in patients with cirrhosis [16]. Previous report demonstrated that the structure of circulating albumin, assessed by HNA, more accurately predicted the outcomes of patients with cirrhosis than serum albumin concentration did [13]. Moreover, supplementation with branched-chain amino acids (BCAA) in cirrhosis nutrition therapy reportedly improves the structure of circulating albumin, i.e., it decreases oxidative albumin levels, and consequently improves mortality rates in patients with cirrhosis [16,17]. These studies suggest that therapies that improve the structure of circulating albumin may prevent complications and improve the prognosis in patients with cirrhosis. However, the effect of HE therapies on the structure of circulating albumin is not well-studied.

Rifaximin is an oral antibiotic with low gastrointestinal absorption and broad-spectrum antibacterial activity [18]. Several studies have reported the beneficial effects of rifaximin on decreasing ammonia levels, improving covert HE, and preventing overt HE recurrence in patients with cirrhosis [18,19]. As hyperammonemia directly induces oxidative stress [10], rifaximin may improve the structure of circulating albumin in patients with cirrhosis by reducing serum ammonia levels. However, whether rifaximin can improve the structure of circulating albumin in patients with cirrhosis remains unclear. Therefore, this study aimed to evaluate the effects of rifaximin on the structure of circulating albumin and serum ammonia levels in patients with liver cirrhosis.

## 2. Materials and Methods

### 2.1. Study Design

This multicenter prospective preliminary study enrolled eight patients with cirrhosis who were treated at Gifu Municipal Hospital (*n* = 4), Gifu Prefectural Hospital (*n* = 2), or Gifu University Hospital (*n* = 2) between July 2019 and December 2021. The study objective and protocol were explained to the patients, and written informed consent was obtained from all participants. Baseline clinical characteristics and laboratory variables were assessed before rifaximin administration. Liver functional reserves were assessed using the Child–Pugh and Model for End-stage Liver Disease (MELD) scores [20,21]. Oral rifaximin (Rifxima^®^ tablet, ASKA Pharmaceutical Co., Ltd., Tokyo, Japan) was administered at a dose of 1200 mg/day, and laboratory data were assessed at 1, 2, and 3 months after initial rifaximin administration. The study protocol was reviewed and approved by the Institutional Review Board of Gifu University (approval number: 2018-054), and the study was performed in accordance with the 1975 Declaration of Helsinki.

### 2.2. Study Population

The study included patients with liver cirrhosis of any etiology with the following characteristics: aged between 20 and 79 years, hyperammonemia (≥80 μg/dL), Child-Pugh score ≤ 9 points, and HE grade I or II according to the West Haven criteria [22]. The diagnosis of cirrhosis was based on clinical, biochemical, and radiological findings. Patients who had been taking rifaximin already, had received liver transplantation, or had an antibiotic allergy, hepatocellular carcinoma, non-hepatic active malignancies, active infection, or life-threatening comorbidities, such as heart failure (New York Heart Association class IV), respiratory failure, and renal failure that required dialysis, were excluded. During the observation period, hepatologists provided interventions such as optimization of energy and protein intake, supplementation with BCAA, late evening snacks, zinc, levocarnitine, and diuretics on the basis of the cirrhosis guidelines [23,24].

### 2.3. Outcomes

The primary outcome of this study was the efficacy of rifaximin therapy on the structure of circulating albumin evaluated by the ratio of oxidized albumin (HNA). As previously reported, HNA was assessed using liquid chromatography/mass spectrometry analyses interfaced with quadrupole-time-of-flight mass spectrometry [13]. The secondary outcomes were changes in the liver functional reserve and serum ammonia levels during rifaximin therapy.

### 2.4. Statistical Analyses

Quantitative variables are expressed as means and standard deviations, and qualitative variables are expressed as numbers. Two groups were compared using the Mann–Whitney *U* test. Time-course changes of quantitative variables were analyzed using the Kruskal–Wallis test, and the Steel test was used for multiple pairwise comparisons. All tests were two-sided, and *p* value < 0.05 was considered significant. Statistical analyses were performed using R software version 4.0.3 (The R Foundation for Statistical Computing, Vienna, Austria).

## 3. Results

### 3.1. Baseline Characteristics of Patients

Among the eight patients, three were men, and the median age and body mass index of all patients were 70 ± 8 years and 26.4 ± 4.4 kg/m^2^, respectively. The etiologies of cirrhosis were hepatitis B (one case), hepatitis C (one case), alcohol-related liver disease (two cases), nonalcoholic steatohepatitis (two cases), and other causes (two cases). The severity of HE was grade I in seven patients and grade II in one. None had ascites, but five patients had esophageal varices. The Child-Pugh class was A in five patients and B in three. Diabetes, hypertension, and dyslipidemia were comorbid in three cases each, and coronary artery disease, atrial fibrillation, and depression were observed in one each. At the time of enrollment, BCAA, nonabsorbable disaccharides, and levocarnitine were administered to five, three, and one patient(s), respectively.

### 3.2. Effect of Rifaximin on Outcomes

**Figure 1:**
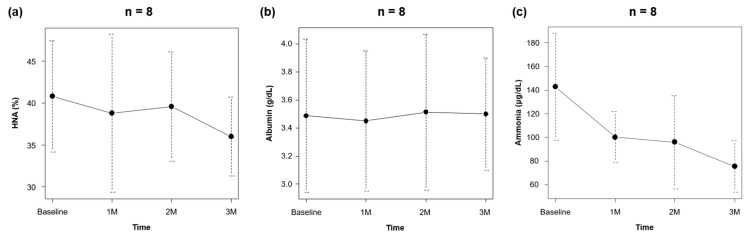
Change in (**a**) HNA and serum (**b**) albumin and (**c**) ammonia levels during rifaximin therapy. Abbreviation: HNA, human nonmercaptalbumin.

The changes in the time-course of each variable at the beginning of rifaximin administration and during treatment are presented in Table 1. At the onset of rifaximin administration, the median Child-Pugh and MELD scores were 6 ± 1 and 11 ± 4, respectively. The median HNA and serum ammonia levels were 41 ± 7% and 143 ± 45 μg/dL, respectively. When the patients were divided into the younger and older group by the median age, there was no difference in HNA (42% vs. 40%; *p* = 0.561) and ammonia (149 μg/dL vs. 136 μg/dL; *p* = 0.885) between two groups. After 3 months of treatment, rifaximin therapy improved HNA from 41% to 36%, although the difference was insignificant (*p* = 0.321; Figure 1a). No significant changes were noted in serum albumin levels (from 3.5 g/dL to 3.5 g/dL, *p* = 1.00; Figure 1b) and liver functional reserve (median Child–Pugh and MELD scores from 6 to 6 and 11 to 11, respectively). After 3 months of rifaximin therapy, serum ammonia levels were significantly decreased from 143 μg/dL to 76 μg/dL (*p* = 0.015; Figure 1c); however, there was no remarkable change in other variables such as the international normalized ratio and levels of white blood cells, red blood cells, hemoglobin, platelets, aspartate aminotransferase, alanine aminotransferase, γ-glutamyl transferase, creatinine, and bilirubin. There was no significant difference in HNA (35% vs. 37%; *p* = 0.885) and ammonia (78 μg/dL vs. 73 μg/dL; *p* = 1.000) between younger and older group after three months of rifaximin therapy.

## 4. Discussion

Albumin is the most abundant plasma protein in humans. Albumin function, determined by cirrhosis-related posttranslational damage, such as albumin oxidation, is more important than albumin concentration in the development of severe HE and mortality in patients with cirrhosis [13]. Traditionally, HNA is considered an oxidative stress marker in patients with cirrhosis [16]. Recent studies focusing on the pathophysiological role of HNA have revealed that HNA has a reduced ability to remove circulating toxic and prooxidant substances [25]. In addition, HNA promotes systemic inflammation by activating immune cells [25]. Therefore, patients with HE may benefit from therapies that improve the structure of circulating albumin. However, evidence on the association between rifaximin therapy and the structure of circulating albumin in patients with cirrhosis is limited, and to our knowledge, this is the first study to investigate the structure of circulating albumin during rifaximin therapy. In this study, our results revealed that rifaximin reduces serum ammonia levels and may improve the structure of circulating albumin by reducing oxidized albumin in patients with cirrhosis. These findings contribute to our understanding of the beneficial effects of rifaximin on the structure of circulating albumin and hyperammonemia in patients with cirrhosis.

Several basic studies have demonstrated the beneficial effects of rifaximin on oxidative stress. Rifaximin prevents indomethacin-induced intestinal damage in rats by reducing tissue inflammation and oxidative stress [26]. It also reduces alcohol-induced oxidative stress by targeting murine hepatic macrophages [27]. Furthermore, recent studies in mice have revealed that combination therapies that include rifaximin maintain intestinal barrier integrity and reduce hepatic lipopolysaccharides, which induce oxidative stress and the progression of liver disease [28,29]. The relationship between rifaximin therapy and oxidative stress in clinical practice, including whether rifaximin acts on the intestinal tract and liver to reduce oxidative stress, is not entirely understood. However, the results of previous studies suggest that rifaximin may have beneficial effects in reducing systematic oxidative stress, at least in part by decreasing oxidized albumin levels in patients with cirrhosis.

The present study also confirmed that rifaximin significantly improves the serum ammonia levels in patients with cirrhosis, as previously reported [19,30]. As hyperammonemia has a negative impact on oxidative stress and HE in patients with cirrhosis [10], the resolution of hyperammonemia may lead to a reduction in oxidative stress. This hypothesis is supported by a previous study that demonstrated that lactulose, an ammonia-lowering agent, alleviates alcohol-induced oxidative stress and neuroinflammation in rats by reducing serum ammonia levels [31]. Our results suggest that rifaximin may improve oxidative stress and the structure of circulating albumin by lowering ammonia levels.

This study had several limitations. First, the small sample size limited the statistical power to prove a significant association between rifaximin therapy and the structure of circulating albumin. Second, our study did not assess other biological oxidative markers or conduct pathological evaluation. Therefore, the association between a change in the structure of circulating albumin and oxidative stress during rifaximin therapy requires further investigation. Third, although dietary intake was optimized based on the Japanese guidelines for cirrhosis [23,24], dietary intake may affect the results of the study. Fourth, since this was a single-arm study without healthy controls or controls without rifaximin therapy, bias and confounding factors may have been introduced. Thus, further large-scale prospective studies are essential to conclude that rifaximin improves the structure of circulating albumin in patients with cirrhosis. Nevertheless, this prospective study may provide valuable insights into rifaximin therapy in patients with cirrhosis.

## 5. Conclusions

In conclusion, to our knowledge, this is the first study to indicate that rifaximin may improve the structure of circulating albumin in patients with cirrhosis. Further studies with larger sample sizes are required to confirm these preliminary results.

## Figures and Tables

**Table 1:** Changes in each variable during rifaximin therapy.

Characteristics	Baseline	1 Month	2 Months	3 Months
Child–Pugh score	6 (±1)	6 (±1)	6 (±1)	6 (±1)
MELD score	11 (±4)	11 (±4)	11 (±4)	11 (±3)
International normalized ratio	1.28 (±0.43)	1.27 (±0.45)	1.22 (±0.26)	1.22 (±0.29)
Platelets (10^9^/L)	76 (±27)	74 (±26)	77 (±33)	75 (±37)
White blood cells (μL)	3130 (±1219)	3256 (±1456)	3070 (±1151)	3386 (±1126)
Red blood cells (10^4^/μL)	336 (±134)	386 (±54)	395 (±49)	399 (±48)
Hemoglobin (g/dL)	12.2 (±1.6)	12.3 (±1.9)	12.2 (±2.0)	12.2 (±2.1)
Creatinine (mg/dL)	0.74 (±0.25)	0.77 (±0.30)	0.78 (±0.27)	0.71 (±0.20)
AST	42 (±16)	44 (±14)	44 (±11)	46 (±17)
ALT	26 (±30)	28 (±11)	29 (±9)	29 (±11)
γ-GTP	82 (±55)	89 (±72)	102 (±105)	119 (±163)
Albumin (g/dL)	3.5 (±0.5)	3.5 (±0.5)	3.5 (±0.6)	3.5 (±0.4)
Bilirubin (mg/dL)	1.7 (±0.5)	1.6 (±0.6)	1.6 (±0.4)	1.8 (±0.5)
Ammonia (μg/dL)	143 (±45)	100 (±22)	96 (±39)	76 (±22) *
HNA (%)	41 (±7)	39 (±9)	40 (±7)	36 (±5)

* *p* < 0.05, compared with baseline. Abbreviations: AST, aspartate aminotransferase; ALT, alanine aminotransferase; GTP, glutamyl transferase; HNA, human nonmercaptalbumin; MELD, model for end-stage liver disease.

## Data Availability

The datasets generated and/or analyzed during the current study are available from the corresponding author upon reasonable request.
